# Pontine infarction isolated to trigeminal sensory fibers: a case report

**DOI:** 10.3389/fpain.2026.1770129

**Published:** 2026-06-03

**Authors:** Lin Wang, Yanliang Niu, Lijun Wang

**Affiliations:** Neurology Department, The Fifth Affiliated Hospital of Zhengzhou University, Zhengzhou, Henan, China

**Keywords:** angioneurotic edema, brainstem infarction, case report, facial numbness, pontine infarction, trigeminal neuropathy

## Abstract

**Background:**

Brainstem infarction involving the trigeminal nerve is not uncommon in clinical practice. However, isolated infarction that selectively damages the sensory fibers of the trigeminal nerve while sparing motor function is extremely rare. Due to its atypical presentation—distinct from complete nerve infarction—this condition is prone to misdiagnosis, posing a significant clinical challenge.

**Case report:**

We report a 59-year-old male with a history of hyperlipidemia, fatty liver disease, and hyperuricemia who presented with progressive swelling and numbness of the left gingiva and face for 20 days. He was initially misdiagnosed with trigeminal neuritis at an external dental clinic and treated with prednisone without symptom relief. Neurological examination revealed decreased pinprick and temperature sensation in the left trigeminal nerve distribution, with normal masseter muscle strength. Brain magnetic resonance imaging showed a diffusion-restricted lesion along the trigeminal sensory pathway in the left pontine tegmentum. A diagnosis of subacute pontine infarction selectively involving the trigeminal sensory fibers was made. Treatment consisted of dual antiplatelet therapy (aspirin 0.1 g at bedtime plus clopidogrel 75 mg once daily), rosuvastatin 20 mg at bedtime, gabapentin 0.3 g three times daily for numbness relief, and loratadine 10 mg once daily for angioneurotic edema. Three-week in-hospital follow-up showed marked improvement of facial swelling and partial relief of numbness.

**Conclusion:**

Isolated infarction of the trigeminal sensory fibers resulting from occlusion of small penetrating pontine arteries is a rare stroke subtype. Early recognition and targeted neuroimaging are crucial for accurate diagnosis and timely intervention.

## 1 Introduction

The trigeminal nerve is the largest mixed cranial nerve and emerges from the pons. The first-order neurons of its sensory fibers are located in the trigeminal (Gasserian) ganglion. Its peripheral branches—the ophthalmic (V1), maxillary (V2), and mandibular (V3) nerves—supply the anterior scalp, facial skin, and the mucous membranes of the eye, nose, and oral cavity. The motor fibers innervate the masticatory muscles and the tensor tympani muscle, mediating chewing and mouth opening. Therefore, damage to the trigeminal nerve typically presents clinically with facial sensory abnormalities and masticatory muscle weakness or paralysis.

Although trigeminal nerve involvement secondary to cerebral infarction is relatively common, pontine infarction selectively affecting only the sensory fibers of the trigeminal nerve is extremely unusual. Cases of pontine infarction involving the entire trigeminal nucleus and its peripheral fibers have been reported, and those patients typically present with trigeminal neuralgia (1–5). To date, only two reported cases resemble the present one, in which facial sensory deficits without trigeminal neuralgia developed after pontine infarction injuring the trigeminal nerve (6, 7). Unlike our case, those two cases had sudden onset upon awakening, whereas our patient experienced progressive symptom development over one week, with facial and gingival swelling as the initial manifestation. Furthermore, multiple sclerosis (MS) is another important cause of isolated pontine lesions, particularly when the lesion involves the trigeminal sensory pathway, presenting with identical facial numbness and sensory disturbances. MS predominantly affects young to middle-aged women, typically shows multiple white-matter demyelinating plaques on FLAIR/T2 MRI, and often responds favorably to glucocorticoid therapy. Therefore, in the differential diagnosis of an isolated pontine trigeminal lesion, ischemic infarction must be rigorously distinguished from a first demyelinating event (clinically isolated syndrome) of MS. Because typical stroke symptoms such as limb weakness, dysarthria, or facial palsy are absent, the diagnosis is often delayed, compromising timely intervention.

This case report aims to highlight the unique diagnostic challenges posed by this rare stroke subtype and to elucidate the pathophysiological mechanisms underlying its atypical presentation—particularly the progressive evolution of symptoms, isolated sensory loss, and accompanying angioneurotic edema—so as to assist clinicians in recognizing and managing similar patients.

## 2 Case description

### 2.1 Medical history and previous treatment

The patient is a 59-year-old Asian male with a past medical history of hyperlipidemia, fatty liver disease, and hyperuricemia (untreated due to absence of symptoms). He underwent “thyroid cystectomy” approximately five years ago. He has no history of hypertension, diabetes mellitus, smoking, or alcohol use, and his family history is unremarkable.

The patient presented with “progressive swelling and numbness of the left gingiva and face for 20 days”. He initially experienced left facial and gingival swelling upon awakening without any identifiable trigger, along with a sensation of bruxism, but did not seek medical attention; the symptoms persisted without relief. Within one week, he gradually developed numbness involving the left half of the face (from the perioral region to the vertex) and nocturnal drooling. Because of pronounced gingival swelling, he visited an external dental clinic. Non-contrast CT of the oral floor, maxillofacial region, and salivary glands showed mild widening of the left submandibular and sublingual ducts, with no evidence of abscess, sialolith, or space-occupying lesion. The dentist made a preliminary diagnosis of trigeminal neuritis or facial neuritis and prescribed mecobalamin and oral prednisone (dose and duration unknown). The patient reported no improvement; instead, he felt that the numbness had slightly worsened. (Notably, the patient's neurological symptoms evolved gradually over several days, rather than reaching a peak within minutes to hours as seen in typical ischemic stroke—this atypical pattern initially steered clinicians toward other diagnoses).

The patient subsequently visited the dental department of our hospital. Oral examination revealed swelling of the left gingiva and buccal mucosa, but no mucosal lesions, ulcers, fistulas, local warmth, tenderness, purulent discharge, or carious pathology. No masses or space-occupying lesions were identified. The dentist therefore referred the patient to the neurology department for further investigation of a neurogenic or neurovascular etiology.

### 2.2 Inpatient work-up

#### 2.2.1 Neurological examination

Neurological examination: The patient was alert, fully oriented, and fluent. Vital signs were stable, with blood pressure 115/85 mmHg. Cranial nerve examination: Selective sensory loss in the left trigeminal nerve distribution—markedly decreased pinprick and temperature sensation over the left cheek, upper lip, lower lip, and gingiva, with partially preserved touch sensation. Masticatory muscle strength was normal, no deviation of the jaw on opening, and the jaw reflex was normal. No facial weakness (symmetrical forehead wrinkles, complete eyelid closure). The remaining cranial nerves were intact (no diplopia, hearing loss, dysphagia, or dysarthria).

Motor system: Normal muscle bulk and tone in all four limbs, strength 5/5, no ataxia or incoordination. No pyramidal or extrapyramidal signs. No hemisensory loss outside the trigeminal distribution. Deep tendon reflexes were symmetric and normal. Bilateral plantar responses were flexor. Gait was steady.

#### 2.2.2 Laboratory investigations

Complete blood count, coagulation profile, liver and kidney function tests, autoantibodies (ANA, ANCA), and serologies for infectious diseases (HIV, syphilis, EBV, CMV) were all normal (Table 1).

**Table 1:** Key laboratory parameters (Table 1).

Parameter	Value	Reference range
Fasting glucose	5.39 mmol/L	3.9–6.1 mmol/L
Total cholesterol	6.4 mmol/L	<5.2 mmol/L
Low-density lipoprotein	4.5 mmol/L	<3.4 mmol/L
Homocysteine	7.9 μmol/L	5–15 μmol/L
Body mass index (BMI)	24.76 kg/m²	18.5–24.9 kg/m²

#### 2.2.3 Imaging studies

Brain magnetic resonance imaging was performed with a comprehensive sequence protocol including T1-weighted, T2-weighted, FLAIR, diffusion-weighted imaging (DWI), and apparent diffusion coefficient (ADC) maps.

##### 2.2.3.1 FLAIR

A focal hyperintensity was noted (Figure 1A). No periventricular or subcortical white matter hyperintensities were seen, arguing against a demyelinating disorder.

**Figure 1:**
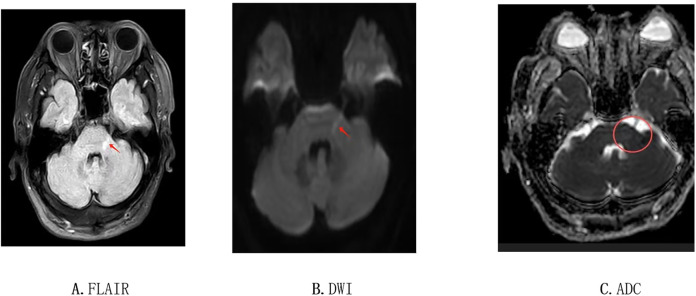
Admission magnetic resonance imaging. Brain MRI findings. **(A)** FLAIR shows a focal hyperintensity in the corresponding region (arrow). **(B)** DWI shows a focal diffusion-restricted hyperintensity along the left pontine tegmentum involving the trigeminal sensory pathway (arrow). **(C)** ADC shows no definite hypointensity within the DWI hyperintense area (circle).

##### 2.2.3.2 Diffusion-weighted imaging (DWI)

A focal hyperintensity was observed in the left pontine tegmentum, along the anatomical course of the trigeminal sensory nucleus and sensory fibers (Figure 1B). The corresponding ADC map showed no hypointensity (Figure 1C), consistent with a subacute infarct. No other diffusion-restricted lesions were present throughout the brain.

##### 2.2.3.3 MR angiography

No significant stenosis or occlusion of the major intracranial vessels; only mild atherosclerotic changes were noted, supporting a small penetrating artery occlusion as the etiology (Figure 1).

### 2.3 Diagnosis and treatment

Based on the acute-to-subacute onset, symptoms strictly confined to the trigeminal sensory fibers with no motor or long-tract signs, presence of vascular risk factors, and MRI confirmation of a focal subacute infarct in the left pontine tegmentum, a diagnosis of **subacute pontine infarction selectively involving the trigeminal sensory fibers** was made, most likely due to occlusion of a small penetrating artery supplying the trigeminal sensory pathway. We infer that the infarction site may be as follows (Figure 2):

**Figure 2:**
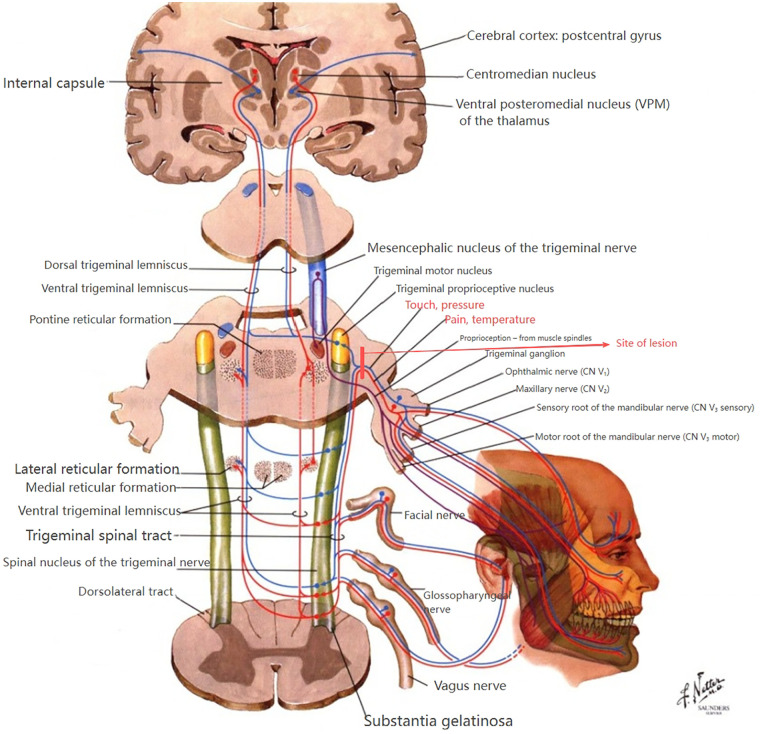
The trigeminal nerve sensory and related sensory systems. Adapted from Netter's Atlas of Human Neuroscience (p. Figure III.8), by D. L. Felten & R. F. Józefowicz, 2006, Elsevier (Singapore) Pte Ltd. Chinese version published by People's Medical Publishing House (30).

#### 2.3.1 Differential diagnosis

- **Multiple sclerosis (MS):** The patient's age (59 years), absence of prior neurological episodes, lack of periventricular or juxtacortical white matter lesions on MRI, and the linear configuration of the DWI lesion (rather than the patchy, ovoid lesions typical of MS) strongly argued against MS. Moreover, the failure of prior prednisone therapy (the patient even felt worsening of numbness) further excluded an inflammatory demyelinating process, which usually responds well to corticosteroids.
- **Trigeminal neuritis:** No prodromal viral syndrome (fever, upper respiratory symptoms, or herpetic lesions), and no nerve thickening on MRI. Lack of response to steroids supported an ischemic rather than inflammatory etiology.
- **Odontogenic or orofacial infection:** Absence of local erythema, warmth, tenderness, purulent discharge, fever, leukocytosis, or elevated inflammatory markers argued against an infectious cause.
- **Ramsay Hunt syndrome:** No periauricular vesicles, otalgia, or lower motor neuron facial palsy.
- **Isolated trigeminal neuralgia:** The predominant symptoms were continuous numbness and swelling rather than paroxysmal electric shock-like pain.

#### 2.3.2 Treatment intervention

After confirming the diagnosis of subacute ischemic stroke, standard secondary prevention was initiated:

- **Antiplatelet therapy:** Aspirin enteric-coated tablets 0.1 g once daily at bedtime plus clopidogrel 75 mg once daily (dual antiplatelet therapy for 21 days, then switched to single agent).
- **Lipid-lowering and plaque stabilization:** Rosuvastatin 20 mg once daily at bedtime.
- **Circulation improvement:** Betahistine injection 30 mg in 0.9% sodium chloride solution for intravenous infusion once daily (dilates cerebral blood vessels: selectively acts on the vertebral artery, basilar artery, and other posterior circulation vessels,increases cerebral blood flow in ischemic areas,improves local blood supply and oxygen delivery,and enhances microcirculation (8–10). It should be noted that in clinical practice across most Western countries, betahistine is not considered a standard treatment for cerebral infarction. This is primarily due to the lack of sufficient evidence from large-scale randomized controlled trials to support its use for ischemic stroke indications. In China, betahistine is used clinically as an adjunctive therapy for cerebral infarction, particularly for symptoms related to posterior circulation ischemia, based on its pharmacological effects of improving microcirculation in the posterior circulation and the inner ear. However, this usage must also comply with the drug label and clinical guidelines in China.)
- **Neuropathic numbness:** Gabapentin capsules 0.3 g three times daily. (Gabapentin binds to the α2δ subunit of voltage-gated calcium channels on neuronal membranes, inhibiting calcium influx and reducing abnormal discharges in peripheral and central sensory pathways (11). Numbness, crawling sensation, burning, hypoesthesia/hyperesthesia, and other paresthesias are manifestations of neuropathic pain, for which gabapentin is a first-line treatment (12, 13).)
- **Angioneurotic edema:** Loratadine tablets 10 mg once daily (empiric antihistamine to reduce swelling).
- **Adjuvant traditional Chinese medicine preparations:** Xuesaitong (notoginseng saponin) injection and Ginkgo biloba extract intravenous infusion (administered as adjunctive “promoting blood circulation and removing stasis” therapy; it is noted that their evidence in acute ischemic stroke is limited, and their use is documented only as part of the treatment record and does not constitute a standard treatment recommendation).

### 2.4 Follow-up and outcome

#### 2.4.1 During hospitalization

On hospital day 2, the patient reported decreased numbness in the lower lip. Facial swelling gradually resolved during the hospital stay. No adverse events occurred.

#### 2.4.2 3-week follow-up

At the outpatient follow-up visit three weeks after discharge, the patient's facial swelling had almost completely resolved (Figure 3), and gingival swelling was no longer prominent. Only mild perioral numbness persisted, which did not affect eating or daily activities. Given the marked improvement in the patient's clinical symptoms, the absence of neurological deficits or other new complaints, and the patient's own refusal to undergo further imaging, no follow-up MRI of the brain or maxillofacial region was arranged after discharge.

**Figure 3:**
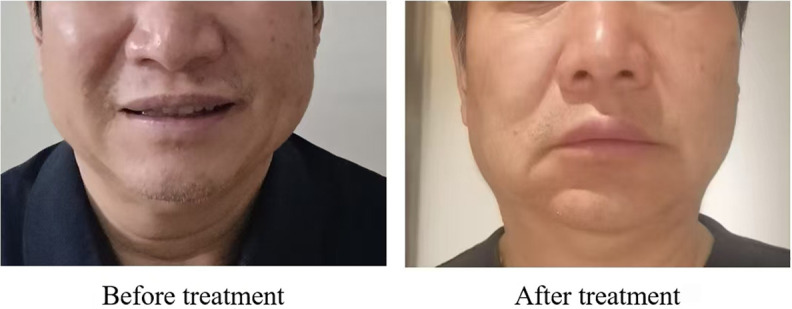
Facial swelling before and after treatment. (Left panel) At admission: swelling of the left facial and perioral soft tissues is visible. (Right panel) Three weeks later: facial swelling has markedly subsided. Due to differences in photograph angle, lighting, and facial expression, the image comparison may not appear striking, but clinical assessment confirmed significant improvement.

### 2.5 Patient history and treatment timeline

The following timeline summarizes key events from symptom onset to post-discharge follow-up (Figure 4).

**Figure 4:**
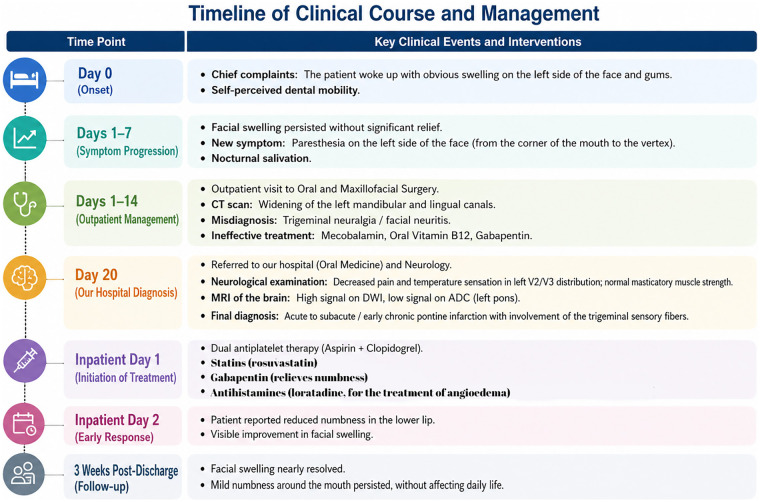
Clinical course timeline: the chart outlines the complete course from initial symptoms through diagnostic evaluation, treatment, and 3-week follow-up.

## 3 Discussion

This case report describes a rare instance of isolated pontine infarction selectively involving the trigeminal sensory fibers, presenting as progressive facial swelling followed by numbness over one week. The clinical presentation was initially misleading, leading the patient to first consult a dentist and receive preliminary diagnoses of trigeminal neuritis and orofacial infection.

### 3.1 Unusual symptom progression

Why did symptoms progress over days rather than peaking within minutes? The symptom evolution in this patient—progressive worsening over one week—is atypical for classic ischemic stroke. Several factors may explain this unusual course:

#### 3.1.1 Vascular territory and mechanism

The affected vessel is a tiny penetrating artery supplying the trigeminal sensory root and the root entry zone. These vessels have extremely poor or absent collateral circulation (14). Ischemia may manifest as progressive hypoperfusion rather than instantaneous complete occlusion, leading to cumulative daily deterioration of neural function rather than sudden loss (15). This “progressive stroke” pattern, although less common in large-artery atherosclerosis, is well documented in small penetrating artery disease of the deep regions or brainstem.

#### 3.1.2 Partial vs. complete nerve injury

The lesion likely did not cause complete necrosis of all trigeminal sensory fibers. Instead, some fibers sustained ischemic functional impairment while others remained structurally intact. Damaged fibers may develop conduction abnormalities and spontaneous ectopic discharges (16), and as ischemic injury accumulates and the inflammatory cascade evolves, symptoms may gradually worsen over the initial days (17). Thus, the patient did not present with complete sensory loss, but rather progressive numbness and swelling—a manifestation of partial, evolving nerve injury rather than immediate complete infarction.

#### 3.1.3 Atypical phenotype masking the classic stroke tempo

Classic stroke presents with *sudden* motor or hemisensory deficits—“alarm symptoms” that prompt immediate medical attention. The phenotype in this case—pure sensory abnormalities confined to a single cranial nerve, accompanied by swelling—was not immediately recognized as a stroke, thereby delaying diagnosis and allowing more time for symptom progression before treatment initiation. The relatively slow progression may also be a combined result of true infarct evolution and delayed presentation.

#### 3.1.4 Reassuringly, a subacute course does not exclude stroke

Although hyperacute (<4.5 h) thrombolysis was not applicable, subacutely progressive small-vessel stroke is well documented (18, 19). The eventual stabilization and improvement of symptoms after antiplatelet and statin therapy confirm the underlying ischemic etiology.

**Clinical takeaway:** In patients with progressive isolated cranial neuropathy, especially those with vascular risk factors, a low threshold for MRI should be maintained even in the absence of typical stroke symptoms.

### 3.2 Pathophysiology of facial swelling

The patient's swelling was objectively present on examination, yet masticatory muscle function was intact, ruling out motor fiber involvement. Several mechanisms may explain this unusual manifestation:

#### 3.2.1 Neurogenic inflammation from neuropeptide release

Trigeminal sensory fibers (especially the V2 and V3 branches) contain peptidergic nerve fibers, including calcitonin gene-related peptide (CGRP) and substance P (20, 21). After infarction, damaged C-fibers release these pre-stored neuropeptides, which act on local orofacial vessels to cause vasodilation and plasma protein extravasation—i.e., neurogenic vasodilatory edema (22). Moreover, surviving sensory fibers may undergo sustained depolarization, leading to continued neuropeptide release and prolonged edema.

#### 3.2.2 Autonomic dysregulation

The trigeminal system has reflex connections with the superior salivatory nucleus (parasympathetic) and the superior cervical ganglion (sympathetic) (23–26). Interruption of trigeminal sensory input may lead to relative parasympathetic predominance and relative sympathetic inhibition, promoting vasodilation, hypersalivation (which could explain nocturnal drooling), and impaired venous return, collectively contributing to edema formation.

#### 3.2.3 Sterile neuroinflammation

Ischemic neurons release damage-associated molecular patterns (DAMPs), activating perivascular macrophages and mast cells, which release pro-inflammatory cytokines that disrupt endothelial tight junctions, further exacerbating edema (27, 28).

Most of the above mechanisms are theoretical, derived from experimental models of trigeminal activation (e.g., migraine models) rather than direct evidence from human post-stroke edema. Nevertheless, they provide a plausible explanatory framework for this unusual presentation.

### 3.3 Clinical implications and teaching points

This case offers several pedagogical lessons. First, it reminds clinicians that acute ischemic stroke can present as a progressive isolated cranial neuropathy, thereby mimicking inflammatory or infectious disorders. Second, it demonstrates that targeted neuroimaging—particularly MRI—is indispensable for establishing the correct diagnosis in such atypical presentations. Third, it shows that even in subacutely progressive stroke, appropriate secondary prevention and symptomatic treatment can lead to meaningful clinical improvement. Fourth, it highlights the importance of multidisciplinary collaboration: although the initial dental evaluation did not identify a dental pathology, the appropriate referral to neurology ultimately led to the correct diagnosis.

### 3.4 Limitations

This report has several limitations. First, no neurophysiological studies (e.g., trigeminal evoked potentials, blink reflex) were performed, which could have provided objective quantitative measures of sensory fiber dysfunction. Second, serum or salivary neuropeptide (CGRP, substance P) levels were not measured; therefore, the proposed neurogenic inflammation mechanism remains indirect inference. Third, the follow-up period was only 3 weeks, and the long-term outcomes of residual numbness and functional recovery are unknown. Fourth, the adjuvant traditional Chinese medicine preparations (Xuesaitong, *Ginkgo biloba* extract) have limited evidence in acute ischemic stroke; their use is documented only descriptively and does not constitute a treatment recommendation. Fifth,the patient presented with left gingival edema on admission,to rapidly relieve clinical discomfort, an empiric antihistamine was initially administered for symptomatic decongestion. Subsequent literature review and mechanistic studies indicate that targeted therapy against the bradykinin pathway—such as plasma kallikrein inhibitors or bradykinin B2 receptor antagonists—is preferred for such edema (29). This is noted here as a supplementary clarification. Finally, as a single case report, the conclusions cannot be generalized; however, the detailed description of this rare presentation may inform future case series or observational studies.

## 4 Conclusion and clinical takeaways

Pontine infarction selectively involving the trigeminal sensory fibers is a rare stroke subtype that can present with subacutely progressive facial swelling and numbness—without pain or motor deficits—and may evolve over days rather than minutes, making it easily misdiagnosed. Key lessons for clinical practice:

- MRI is essential for any progressive isolated cranial neuropathy, especially in patients with vascular risk factors.
- Exclude multiple sclerosis (MS) when facing an isolated pontine lesion: MS typically shows ill-defined or multifocal demyelination on FLAIR/T2, occurs in younger women, and responds well to steroids—distinct from our older, steroid-unresponsive patient with vascular risk factors and characteristic MRI findings.
- Gabapentin can relieve post-stroke numbness even without pain, targeting ectopic neural discharge.
- Timely neuroimaging and secondary prevention (antiplatelet, statin, and symptomatic therapy) lead to favorable outcomes despite an atypical presentation.

This case underscores the need to systematically rule out MS in isolated pontine lesions and provides a practical reference for recognition and management of similar patients.

## 5 Patient perspective (expressed in the patient's own first-person voice)

“When my face first started swelling, I thought it was a dental problem. I went to the dentist, but the medicine they gave me didn’t work, and the numbness gradually spread. I was very worried. After I was referred to the neurology department and had an MRI, I finally learned that I had had a small stroke. The doctor explained why my face became swollen and numb. After treatment, the swelling went away quickly. The numbness in my lip is still a little bit present, but it's much better. I am very grateful to have received the correct diagnosis and treatment. I agree to share my story because it might help other patients with similar symptoms get the right diagnosis earlier”.

## Data Availability

The raw data supporting the conclusions of this article will be made available by the authors, without undue reservation.
